# Peritonitis as the first presentation of systemic lupus erythematous: a case report

**DOI:** 10.1186/s13256-021-03216-3

**Published:** 2021-12-26

**Authors:** Zahedin Kheyri, Amirreza Laripour, Moein Ala

**Affiliations:** 1 Baharloo Hospital, Tehran University of Medical Sciences (TUMS), Tehran, Iran; 2 Department of Gastroenterology, Tehran University of Medical Sciences (TUMS), Tehran, Iran; 3 Students’ Research Committee, Arak University of Medical Sciences, Arak, Iran; 4 School of Medicine, Tehran University of Medical Sciences (TUMS), Tehran, Iran

**Keywords:** Systemic lupus erythematous, Peritonitis, Ascites, Low-gradient high-protein ascites fluid, Case-report

## Abstract

**Background:**

Systemic lupus erythematosus is an inflammatory disease affecting several organs. Serositis is one of the systemic lupus erythematosus presentations, but peritonitis is a relatively rare presentation. Particularly, it is extremely rare to observe peritonitis as the first presentation of systemic lupus erythematosus.

**Case presentation:**

Here, we present a case of peritonitis without other symptoms of systemic lupus erythematosus, in a patient who was finally diagnosed with systemic lupus erythematosus. Our patient was a 27-year-old Persian/Caucasian male with fatigue, weakness, weight loss, abdominal distension, massive ascites, and normocytic hemolytic anemia. He did not mention any prior medical conditions and did not use any drugs. There were no signs of thyroid dysfunction, cardiac dysfunction, cancers, infectious diseases, hepatitis, kidney diseases, or other diseases. Low-gradient, high-protein ascites fluid, and positive antinuclear antibody and anti-double stranded DNA were in favor of systemic lupus erythematosus. Corticosteroid pulse therapy led to resolution of ascites, and the patient was discharged with prednisolone and hydroxychloroquine.

**Conclusion:**

Peritonitis is a rare presentation of systemic lupus erythematosus, particularly as the first presentation and in the absence of other signs and symptoms; however, systemic lupus erythematosus should be considered as one the differential diagnoses for peritonitis when other etiologies have been ruled out.

## Background

Systemic lupus erythematosus (SLE) is a chronic, multi-organ inflammatory disease that involves the joints, skin, cardiovascular system, lungs, central nervous system, and kidneys [1]. SLE peritonitis is one of the rarest manifestations of SLE, and presents as painless ascites, concurrent with other known symptoms of SLE [2, 3]. The occurrence of SLE ascites is typically associated with nephrotic syndrome, protein-losing enteropathy, and constrictive pericarditis. The occurrence of ascites and peritonitis as primary manifestations of SLE is extremely rare [4].

In this case report, we present a young man with ascites and lupus peritonitis as the first manifestations of SLE.

## Case presentation

The patient was a 27-year-old Persian/Caucasian man with no medical history, who experienced 2 months of abdominal distension and weakness, with concurrent 10 kg unintentional weight loss. He did not mention nausea, vomiting, dysphagia, diarrhoea, cough, dyspnea, night sweats, or chills. He had no gastrointestinal complaints except feeling fullness. The patient was normoglycemic and had no history of hepatitis, jaundice, diabetes, drug abuse, or alcohol use. On presentation, his blood pressure was 120/80 mmHg and his body temperature was 37 °C. Abdominal ascites was apparent, with no icterus or edema. There were no signs of cheilosis, glossitis, lymphadenopathy, or organomegaly on physical examination. Serological tests for viral hepatitis and human immunodeficiency virus (HIV) were negative.

Laboratory findings are presented in Table 1.

**Table 1:** Laboratory findings at admission

Variable	Result	Variable	Result
White blood cell count (WBC)	10,800/μL (4500 < NL < 11,000)	Aspartate transaminase (AST)	14 IU/L (NL < 40)
Neutrophil	74%	Alanine transaminase (ALT)	10 IU/L (NL < 40)
Lymphocyte	22%	Alkaline phosphatase (ALP)	102 IU/L (NL < 147)
Hemoglobin	8.5 g/dL (14 < NL < 18)	Total bilirubin	0.7 mg/dL (NL < 1.2)
Mean corpuscular volume (MCV)	85 fL (80 < NL < 100)	Blood urea nitrogen (BUN)	16 mg/dL (6 < NL < 24)
Platelet count	301,000/μL (150,000 < NL < 450,000)	Creatinine	0.7 mg/dL (NL < 1.2)
Albumin	4 g/dL (3.5 < NL < 5.4)	Total protein	8 g/dL (6 < NL < 8.3)

The patients reticulocyte production index was more than 2.5. The 24-hour urine test detected 250 mg of protein. Color Doppler ultrasonography of abdominal vessels was normal. Abdominal, pelvic, and chest computed tomography (CT) scan showed bilateral pleural effusions and massive ascites. The patient underwent abdominal paracentesis, which revealed a low-gradient, high-protein fluid. Ascites fluid adenosine deaminase (ADA) and QuantiFERON test results were negative. In addition, PCR test was negative for tuberculosis. Furthermore, cytological test showed no evidence of malignancy. No signs of cardiac dysfunction were detected in his echocardiography. In addition, his thyroid function test, upper gastrointestinal endoscopy, and chest X-ray were normal.

Diagnostic laparoscopy and peritoneal biopsy were also negative for malignancy. With the exclusion of other etiologies of ascites, rheumatologic tests were requested. His ANA and anti-dsDNA were reported high. Due to positive antinuclear antibodies (ANA) and anti-dsDNA, as well as low levels of complement C3 and C4, the patient was diagnosed with lupus peritonitis. Initially, he was treated with methylprednisolone pulse therapy (1000 mg/day for three consecutive days) and then with high-dose prednisolone of 60 mg/day, which was finally tapered to 7.5 mg/day. The initial corticosteroid pulse therapy markedly improved ascites, and the patient was discharged with prednisolone and hydroxychloroquine. He was followed-up in the outpatient clinic up to 3 months. His symptoms did not return up to 3 months. He was not accessible after 3 months of follow-up.

## Discussion

SLE is an autoimmune, multiorgan disease [5]. One of the American College of Rheumatology diagnostic criteria for SLE is pleural and pericardial inflammation, which is nonspecific but prevalent [6, 7]. Serous membrane inflammation involving the pericardium, pleura, and peritoneum can cause pain, fluid accumulation, adhesions, and even fibrosis [2]. A European study with a 10-year analysis of 1000 patients with SLE follow-up found that serositis occurred in nearly 16%; however, peritoneal involvement was extremely rare [8].

In an autopsy study of patients with SLE, 60–70% had peritoneal involvement, whereas only 10% showed clinical manifestations throughout their lives [9]. Ascites in SLE is rarely massive; it is often painless, has a gradual onset, and is rarely reported as the first manifestation of SLE, usually following other symptoms. In addition, ascites can be a result of nephrotic syndrome, protein-losing enteropathy, or constrictive pericarditis [10]. However, ascites appeared as the first presentation of SLE in our patient.

The first step in ascites treatment is the correct diagnosis of its etiology. Portal hypertension and peritoneal disease can lead to fluid accumulation in the abdominal cavity through activation of the renin–angiotensin–aldosterone system, sodium and water retention, increased plasma volume, and fluid secretion into the serosa-lined cavity [11].

The first step in diagnosing the etiology of ascites is fluid analysis. In portal hypertension, ascites results from vascular hydrostatic pressure, which leads to the formation of transudate fluid. On the other hand, in inflammatory or neoplastic peritoneal disease, changes in the permeability of vascular membranes result in the production of protein-rich exudative fluid [11].

The mechanism of ascites development in lupus peritonitis has not been fully identified; however, there are two theories. One is the production of autoantibodies by B-lymphocytes. These autoantibodies precipitate in the peritoneum and cause local inflammatory reactions through binding to antigens and forming immune complexes. Another theory attributes ascites to inflammation of peritoneal vessels or serous membranes surrounding abdominal organs. Due to peritoneal involvement in patients with SLE in both conditions, ascites fluid is exudative [12].

Lupus peritonitis should be considered in the differential diagnosis of patients with exudative ascites after exclusion of other more common causes, including peritoneal carcinomatosis, primary mesothelioma, peritoneal pseudomyxoma, hepatocellular carcinoma, peritoneal tuberculosis, HIV-associated peritonitis, nephrotic syndrome, protein-losing enteropathy, and severe malnutrition [11]. Our patient had massive ascites without typical symptoms of SLE as defined by the American College of Rheumatology. During his workup, there were no findings in favor of other more common etiologies of ascites, and his laboratory findings were suggestive of SLE. Despite previous studies, this rare case of SLE shows that SLE can present with massive ascites as the first presentation. The absence of other clinical findings cannot completely rule out SLE and it should be considered as one of the differential diagnoses.

The prognosis of lupus peritonitis is usually good when appropriate treatment is initiated. The treatment is based on nonsteroidal antiinflammatory drugs (NSAIDs) and corticosteroids. In refractory cases characterized by persistent fluid accumulation in the serosal cavity, immunomodulators or immunosuppressants, particularly pulse therapy with cyclophosphamide, can be used [2, 8, 10, 13]. Similarly, our patient responded to initial corticosteroid pulse therapy and his ascites volume decreased within a few days.

## Conclusion

Serositis is a common finding in SLE; however, peritoneal involvement is extremely rare, particularly as the first presentation of the disease. SLE peritonitis should be considered in the differential diagnosis of patients with exudative ascites after exclusion of other causes. This rare case of SLE shows that peritonitis can be the first clinical presentation of SLE, and the absence of typical symptoms of SLE cannot rule out the disease. The prognosis of SLE peritonitis is usually good, and treatment is based on NSAIDs and corticosteroids. Immunomodulators and immunosuppressants should be considered for refractory SLE peritonitis.

## Data Availability

Not applicable
